# The role of meningioma integrated molecular profiling to improve patient management and disclose novel therapeutic targets

**DOI:** 10.1093/braincomms/fcaf088

**Published:** 2025-02-23

**Authors:** Luca Bertero, Marta Padovan, Giuseppe Lombardi

**Affiliations:** Pathology Unit, Department of Medical Sciences, University of Turin, 10124 Turin, Italy; Department of Medical Oncology, Oncology 1, Veneto Institute of Oncology IOV-IRCCS, 35128 Padua, Italy; Department of Surgery, Oncology and Gastroenterology, University of Padua, 35128 Padua, Italy; Department of Medical Oncology, Oncology 1, Veneto Institute of Oncology IOV-IRCCS, 35128 Padua, Italy

## Abstract

This scientific commentary refers to ‘Integrating genome-wide association studies and transcriptomics prioritizes drug targets for meningioma’, by Liao *et al*. (https://doi.org/10.1093/braincomms/fcaf053).

**This scientific commentary refers to ‘Integrating genome-wide association studies and transcriptomics prioritizes drug targets for meningioma’, by Liao *et al*. (** https://doi.org/10.1093/braincomms/fcaf053 **).**

Meningiomas are the most frequent primary central nervous system tumour,^1^ and even though most of the cases show an indolent course which is consistent with a World Health Organization (WHO) Grade 1 neoplasm, they can significantly impair patients’ quality of life and/or outcome. Meningiomas can hamper patients’ health due to a combination of factors including the specific location (e.g. skull base) which can impede a complete surgical resection and/or their intrinsic biological aggressiveness. For these reasons, molecular characterization of meningiomas has been a significant focus of neuro-oncological research during the last decade. The ultimate aims of these efforts are to improve meningioma prognostication, design tailored treatment algorithms and identify novel therapeutic targets.

Initially, research has been focused on the identification of pathogenic genetic alterations like single nucleotide variations or small insertions and deletions, thus allowing to disclose several associations between the specific molecular traits and a wide range of tumour characteristics like location, histopathological type and WHO grade, association with tumour predisposition syndromes and outcome.^2^ Secondly, the characterization of the meningioma epigenetic landscape has been another fruitful endeavour. Nowadays, tumour DNA methylation profiling is a critical diagnostic tool in neuropathology, and focusing on meningiomas, it has allowed to identify multiple subgroups which have been found to be more faithfully associated with prognosis compared with conventional histopathological grading.^3^ Even more recently, multiomic approaches including DNA sequencing, transcriptomic analyses, epigenetic profiling and proteomic characterization have been applied to meningioma cohorts to further define clinically relevant subgroups.^4,5^ For example, Nassiri *et al*.^4^ proposed four groups characterized by different signatures including immune regulation (MG1), angiogenesis (MG2), hypermetabolism (MG3) and cell-cycle (MG4), while Choudhury *et al.*^6^ proposed the three immune-enriched, merlin-intact and hypermitotic subgroups.

In their recent study published in *Brain Communications*, Liao *et al*.^7^ leveraged on multiple molecular datasets, including genome-wide association studies and transcriptomic profiling, to search out for genes potentially significant for meningioma oncogenesis. The identified genes, *XBP1*, *TRPC6* and *TTC28*, are associated with multiple functions including endoplasmic reticulum cellular stress response and immune regulation, calcium-based intracellular signalling pathways and cell-cycle, respectively. Study findings also suggest the relevance of these genes’ products for tumour microenvironment modulation. Regarding the therapeutic relevance of the genes identified by Liao *et al*.,^7^ potential clinically relevant inhibitors for *TTC28* and *TRPC6* have been identified based on docking analysis.

Immune regulation has been found to be a critical hallmark for the MG1 group identified by Nassiri *et al*.^4^ corresponding to the immune-enriched group by Choudhury *et al.*^6^ and the MenG B group by Bayley *et al*.^5^ In particular, these meningiomas are mostly restricted to WHO Grades 1 and 2 and show a higher immune cell infiltration as well as increased expression of *HLA* genes and of signatures related to extracellular matrix remodelling and presence of lymphatic vessels. In terms of molecular alterations, these meningiomas are associated with *HLA* locus gain on chromosome 6p and *NF2* locus loss paired up with *NF2* gene mutations. Conversely, increased expression of cell-cycle-related signatures has been observed in the MG4 proliferative group by Nassiri *et al*.,^4^ the hypermitotic proliferative group by Choudhury *et al.*^6^ and the MenG C group by Bayley *et al*.^5^ This group showed a downregulation of pathways associated with immune response matched with an enrichment of proliferation-associated transcription factors. This subset is also linked with poorer outcomes and a higher rate of WHO Grade 3 meningiomas. Frequent copy number alterations are also observed including chromosome 1p loss and *NF2*, *HLA* and *CDKN2A/B* loci losses.

Focusing on the potential therapeutic targeting of the disclosed molecular hallmarks, Liao *et al*. observed a potential relevance of glucocorticoid-based treatment with dexamethasone to decrease expression of the *TRPC6* and *TTC28* genes. This finding is of interest considering the observation by Choudhury *et al.*^6^ that merlin expression, the product of the *NF2* gene, contributes to the preservation of a low apoptotic threshold in the merlin-intact group, due to the inhibition of glucocorticoid receptor expression through modulation of ARHGAP35, a DNA binding factor. Increased sensitivity to apoptosis could thus translate into higher susceptibility to antineoplastic cytotoxic treatment.

Possible therapeutic targets for meningiomas include NF2, mTOR, PIK3CA, PDGFR, AKT, somatostatin receptor (SSTR), smoothened (SMO), cyclin-dependent kinases and inhibitors (CDKN2A/B, CDK4, CDK6), progesterone receptor and oestrogen receptor and VEGF/VEGFR.

SSTR-targeted therapies, such as peptide receptor radionuclide therapy, have promising potential. The radioligand [177Lu]Lu-DOTATATE is currently being investigated in a randomized clinical trial from EORTC (LUMEN-1, NCT06326190) in SSTR2-positive meningiomas. Bevacizumab, a VEGF inhibitor, has shown some benefit in progression-free survival; however, its efficacy is based on retrospective series and case reports.

New agents are currently being investigated in clinical trials, offering hope for advancing treatment options for meningiomas: among them, regorafenib (MIRAGE trial, NCT06275919), apatinib (NCT04501705), ribociclib (NCT02933736), selumetinib (NCT03095248), trametinib in combination with alpelisib (NCT03631953) and immune-checkpoint inhibitors (NCT02648997).^8^

Furthermore, umbrella trials can overcome several challenges, including disease heterogeneity and the lack of standardized outcome measures. The Alliance/NCI A071401 study is a genomically driven Phase 2 trial that serves as a key example. This trial evaluates various drugs, including the FAK inhibitor GSK2256098, the CDK4/6 inhibitor abemaciclib, the SMO inhibitor vismodegib and the AKT inhibitor capivasertib (AZD5363). In the FAK inhibitor arm, 37 patients were enrolled. The study successfully met its 6-month progression-free survival endpoints demonstrating excellent tolerability.^9^

As acknowledged by the authors, even though novel data regarding potential therapeutic targets is surely of interest, caution is warranted. As discussed, meningiomas represent a pleomorphic tumour group, consisting of several subgroups with significant differences based on clinico-pathological and molecular characteristics. Thus, analysis of limited datasets, although useful for exploratory analyses, has to be validated in larger cohorts, possibly representative of the most clinically relevant samples. Functional validation is also necessary to support the relevance of the novel candidate therapeutic targets and to identify reliable predictive markers. As has been previously shown,^4,6^ sensitivity to specific treatment approaches is dependent on the meningioma subgroup, thus the development of reliable and accessible predictive markers is critical to translate the collected data into effective treatments. Moreover, although the availability of multiple, comprehensive and independently generated datasets is surely welcome, efforts should also be aimed at integrating the newly acquired data in well-defined consensus groups to increase results reproducibility. This effort is ongoing and a consensus document has been recently published by the International Consortium on Meningiomas.^10^

## Competing interests

L.B. has received honoraria for lectures from Servier. M.P. reports a relationship with consulting or advisory role funding from Novocure, Bayer and Helath4U. G.L. reports a relationship with consulting or advisory role funding from ABBVIE, Bayer, Novartis, Orbus Therapeutics, BrainFarm, Celgene, CureTeq, GlaxoSmithKline, Health4U, Braun, Janssen, BioRegio Stern, Servier, Novocure and travel funding from Roche and Bayer, Servier.
